# DCE-MRI Pharmacokinetic-Based Phenotyping of Invasive Ductal Carcinoma: A Radiomic Study for Prediction of Histological Outcomes

**DOI:** 10.1155/2018/5076269

**Published:** 2018-01-17

**Authors:** Serena Monti, Marco Aiello, Mariarosaria Incoronato, Anna Maria Grimaldi, Michela Moscarino, Peppino Mirabelli, Umberto Ferbo, Carlo Cavaliere, Marco Salvatore

**Affiliations:** ^1^IRCCS SDN, Naples, Italy; ^2^Department of Pathology, Ospedale Moscati, Avellino, Italy

## Abstract

Breast cancer is a disease affecting an increasing number of women worldwide. Several efforts have been made in the last years to identify imaging biomarker and to develop noninvasive diagnostic tools for breast tumor characterization and monitoring, which could help in patients' stratification, outcome prediction, and treatment personalization. In particular, radiomic approaches have paved the way to the study of the cancer imaging phenotypes. In this work, a group of 49 patients with diagnosis of invasive ductal carcinoma was studied. The purpose of this study was to select radiomic features extracted from a DCE-MRI pharmacokinetic protocol, including quantitative maps of *k*^trans^, *k*_ep_, *ve*, iAUC, and *R*_1_ and to construct predictive models for the discrimination of molecular receptor status (ER+/ER−, PR+/PR−, and HER2+/HER2−), triple negative (TN)/non-triple negative (NTN), ki67 levels, and tumor grade. A total of 163 features were obtained and, after feature set reduction step, followed by feature selection and prediction performance estimations, the predictive model coefficients were computed for each classification task. The AUC values obtained were 0.826 ± 0.006 for ER+/ER−, 0.875 ± 0.009 for PR+/PR−, 0.838 ± 0.006 for HER2+/HER2−, 0.876 ± 0.007 for TN/NTN, 0.811 ± 0.005 for ki67+/ki67−, and 0.895 ± 0.006 for lowGrade/highGrade. In conclusion, DCE-MRI pharmacokinetic-based phenotyping shows promising for discrimination of the histological outcomes.

## 1. Introduction

Breast cancer is the most common malignant tumor that affects women worldwide [1, 2]. It is one of the leading cause of cancer death in women, with alarming statistics in the young population (under 40 years) [3].

An early diagnosis and classification of the breast tumor is fundamental in the patient's management: the tumor genotype is often predictive of outcome [4], it is used clinically for the selection of the most appropriate therapy [5–7] and has proved valuable for personalized treatments [8–10].

According to their gene expression, breast tumors can be classified into four molecular subtypes: luminal A (lumA), luminal B (lumB), human epidermal growth factor receptor 2- (HER2-) like, and basal-like [11]. This classification is based on the expression of estrogen receptor (ER), progesterone receptor (PR), HER2, and ki67, a marker of cellular proliferation. According to St. Gallen 2013 [12], the lumA subtype, defined as positive ER (ER+), positive PR (PR+, with a positive value larger than 20%), negative HER2 (HER2−), and low levels of ki67 (with a cut off of 20% [13]), shows the best survival and the highest probability of being long-term disease-free [14]. LumB subtypes are characterized by two different genotypes: ER+ combined with HER2− and PR < 20% or high levels of ki67 (≥20) and ER+ together with HER2+ with any value of PR and ki67. LumB has higher proliferation and poorer prognosis than lumA [15]. HER2 (negative to ER and PR, positive to HER2) and basal-like (negative for all three receptors and, consequently, also known as triple negative, TN) subtypes have the worst prognosis and the latter is often associated with lymph node involvement [16] and accounts for a large portion of breast cancer deaths after diagnosis [17]. These receptors together with ki67, providing direct observation on the molecular underpinnings of the tumor, have been widely studied and are at the basis of the choose for personalized treatments: for example, patients with HER2+ cancer have been found to be quite effectively treated with trastuzumab and lapatinib [18]; ki67 has been identified as a prognostic and predictive marker in hormone receptor positive breast cancer [19]. Breast cancers overexpressing ER, PR, and/or HER2 can be specifically targeted with hormonal therapies, while TN breast cancers currently have no targeted therapy available and are limited to general cytotoxic chemotherapies [20].

Another important clinical variable for patients' stratification and treatment options is the tumor grade. For breast cancer, it is defined by the Elston-Ellis modification of the Scarff-Bloom-Richardson grading system and it is based on duct structures, size, and shape of nucleus in the tumor cells, and mitotic rate, leading to a final three-grade scale: G1 (low grade), G2 (intermediate grade), and G3 (high grade), with lower grade indicating a better prognosis [21].

The molecular receptor status, ki67 levels, and tumor grade are obtained by immunohistochemical analyses on tissue samples [22] from core needle biopsy (CNB). CNB is widely used as a standard procedure for diagnosis of breast cancer [23], but, although several studies have reported the concordance between preoperative CNB and surgical specimens for molecular determination [24, 25], it has two main limits. CNB, in fact, is an invasive procedure and it may not reflect completely the complexity and heterogeneity of tumor lesion, since the information obtained may vary depending on which part of the tumor is sampled [26].

In recent years, an increasing interest has been focused on the identification of imaging surrogates and development of noninvasive diagnostic tools for cancer characterization and monitoring [27]. In fact, the imaging approach, besides its noninvasiveness, can give in vivo information on the entire tumor volume, reducing inaccuracy due to sampling errors in histopathological analyses. In particular, radiomic approaches have proved to be a key way to study the cancer imaging phenotypes, reflecting underlying gene expression patterns [28, 29]. Radiomics, in facts, refer to the extraction of a large number of quantitative features from medical images [30], revealing heterogeneous tumor metabolism and anatomy [31, 32]. This high-throughput extraction is preparatory to a process of data mining [33] for studies of association with or prediction of different clinical outcomes [34], giving important prognostic information about disease. The potential of radiomics, to extensively characterize the intratumoral heterogeneity, has shown promise in the prediction of treatment response and outcome, differentiating benign and malignant tumors and assessing the relationship with genetics in many cancer types [35], such as non-small-cell lung cancer [36], liver [37], prostate [38] and head and neck [39] tumors, and glioblastoma [40]. In the last years, the most widely used imaging modalities in radiomic research have been positron emission tomography (PET) and computed tomography (CT) [41]; however, an increasing interest is emerging toward Magnetic Resonance Imaging (MRI), which is an extremely versatile imaging technique, as it can provide multiparametric information derived from both morphologic and functional signals [42].

In particular, in breast cancer research, several radiomic studies have been performed and are mainly based on dynamic contrast-enhanced- (DCE-) MRI or combine MRI with other imaging modalities, such as PET [43]. MRI, in fact, is the most sensitive imaging modality for soft tissue tumor detection, characterization, and accurate extent definition [14, 44]; moreover, DCE-MRI is of great value in the characterization of anatomic and functional properties of breast cancer [45]. Previous radiomic studies of breast cancer have been conducted for invasiveness evaluation [46, 47], treatment response [48–50] and recurrence [51, 52] prediction, and genomic correlation [51], but the majority is focused on the differentiation between molecular subtypes [14, 16, 20, 34, 44, 52–58].

Although all of them are based on the extraction of morphological features and enhancement features from DCE-MRI, no one takes into account the radiomic evaluation of the quantitative DCE pharmacokinetic parameters (*k*^trans^, *k*ep, iAUC, and *ve*) that, in standard correlation analysis with mean values, have shown a good agreement with prognostic factors and TN subtypes [59]. To overcome this approach and take into account lesion heterogeneity, Li et al. [60] performed a histogram-based analysis for differentiating benign and malignant tumors.

The aim of this study was to select radiomic features extracted from a DCE-MRI protocol, including precontrast images, pharmacokinetic parametric maps, the auxiliary *R*_1_ map, and delayed postcontrast images, to evaluate their prediction power in the differentiation of molecular receptor status, ki67 levels, and tumor grade obtained by immunohistochemical analyses in a dataset of invasive ductal carcinoma patients.

## 2. Materials and Methods

### 2.1. Patient Cohort

The study was approved by the Institutional Review Board. A group of 49 patients was enrolled. Inclusion criteria were diagnosis of invasive ductal carcinoma, availability of the core biopsy or mastectomy biopsy reports of the primary breast cancer, and age older than 18 years at the time of the study. Exclusion criteria included pregnancy and inadequate MR images.

### 2.2. MR Imaging

MR examinations were performed on a 3T Biograph mMR (Siemens Healthcare, Erlangen, Germany) with a dedicated 4-channel breast coil. The imaging protocols included a Turbo inversion recovery magnitude (TIRM) sequence (TR = 4200 ms, TE = 60 ms, TI = 230 ms, FOV = 380 × 380 mm^2^, resolution = 1.48 × 1.48 mm^2^, and slice thickness = 4 mm); 6 gradient echo volumetric interpolated breath-hold examination (VIBE) sequences at variable flip angle (FA) for T1 mapping (TR = 5.3 ms, TE = 1.9 ms, FAs = [2°, 5°, 8°, 12°, 15°, 20°], FOV = 356 × 379 mm^2^, resolution = 1.98 × 1.98 mm^2^, and slice thickness = 3.6 mm); a dynamic scan with 60 consecutive phases with a VIBE sequence (TR = 5.3 ms, TE = 1.9 ms, FA = 20°, FOV = 356 × 379 mm^2^, resolution = 1.98 × 1.98 mm^2^, slice thickness = 3.6 mm, and temporal resolution = 9 s/phase); and a delayed 3D postcontrast fat-suppressed T1-weighted gradient echo sequences (TR = 8.4 ms, TE = 2.5 ms, FOV = 370 × 370 mm^2^, resolution = 0.82 × 0.82 mm^2^, and slice thickness = 0.89 mm). Intravenous contrast injection started at the end of the first phase of dynamic scan at a dose of 0.1 mmol/kg of body weight and at the highest rate compatible with patient's age and compliance (up to 5 mL/s)

### 2.3. Immunohistochemistry

Core needle biopsies were performed under ultrasound guidance by a radiologist with more than 15 years of experience. Biopsies were fixed in 10% neutral buffered formalin at the time of biopsy. Mastectomy specimens, obtained from patients who underwent mastectomy, were sent to the department of pathology immediately after resection. Expression of ER, PR, HER2, and Ki67 was determined by immunohistochemical analysis. Each tumor sample was classified as ER+, PR+, and/or HER2+, or being TN. The cut-off values for receptor and ki67 expression were chosen accordingly to the St Gallen Consensus Meeting 2013 [12]. The histological grade was determined using the method of Elston and Ellis. All pathological diagnoses were rendered by the Breast Pathology Subspecialty Department at Ospedale Moscati (Avellino, Italy).

### 2.4. Tumor Segmentation

3D segmentation of the lesion was obtained semiautomatically from the dynamic VIBE sequence. After motion correction of the single phases on the first time point, an experienced radiologist was asked to manually draw a rectangular bounding box containing the tumor region. Successively, the dynamic motion-corrected sequence and the bounding box were given as input to the SegmentCAD module of 3DSlicer [61], which automatically segmented the lesion on the basis of the temporal dynamic of the signal. Voxels that reached a signal increase higher than the 75% of the first time point were selected as tumor. The cut-off of 75% was selected in accordance with a previous study [62] that studied the concordance correlation coefficient between the longest dimensions of the tumor measured on the surgical specimen and on the DCE-MRI segmentation when the cut-off value changed.

### 2.5. Pharmacokinetic Map Calculation

Pharmacokinetic maps were obtained with the commercial software Tissue 4D (Siemens Healthcare, Erlangen, Germany). After an automated step of motion correction of the VIBE sequences at variable FAs with the dynamic VIBE sequence, the Toft model [63] was chosen for the pharmacokinetic parameters calculation. The arterial input function (AIF) used for the analysis was set to “intermediate,” on the basis of population-based AIFs built in Tissue 4D. Finally, 3D maps of *k*^trans^, *k*_ep_, *ve*, and iAUC were obtained.

In addition to these quantitative maps, from the fitting of the VIBE signal at variable FAs also the relaxation rate *R*_1_ (inverse of relaxation time *T*_1_, used in the generation of pharmacokinetic parameters) was obtained, by an in-house software developed in Matlab (The MathWorks Inc., Natick, MA) and saved for feature extraction.

### 2.6. Image Preprocessing

Before feature extraction, some preprocessing steps were performed: for each subject, in order to avoid the presence of spurious points in the tumor masks, possible voxels, disconnected from the biggest connected component, were erased. Then, the TIRM and the delayed 3D postcontrast fat-suppressed *T*_1_-weighted (postC) images were coregistered to the first time point of the dynamic VIBE sequence in order to correct for possible patients' movements and two resampled versions of the tumor mask were generated, in order to match the resolution of TIRM and postC images and to allow feature extraction in the native space, for each acquisition. This step was not required for *R*_1_ map, since it shared the same geometry of dynamic VIBE sequence, which was used for lesion segmentation, and consequently of *k*^trans^, *k*_ep_, *ve*, and iAUC maps.

### 2.7. Feature Extraction

Nine shape features (including number of voxels, maximum and minimum diameter, volume, surface area, surface volume ratio, compactness, spherical disproportion, and sphericity) were extracted from the tumor segmentation.

*R*_1_, *k*^trans^, *k*_ep_, *ve*, and iAUC maps and TIRM and postC were used for first- and second-order feature extraction. They were normalized, limiting their dynamics within the tumor mask to *μ* ± 3*σ* [64]; then thirteen first-order features were extracted from the intensity histogram computed on 256 bins: energy, entropy, kurtosis, maximum (Max), mean, mean absolute deviation (Mad), median, minimum (Min), root mean square (Rms), skeweness, standard deviation (Std), uniformity, and variance.

The second-order features chosen for this study were Gray Level Cooccurrence Matrix (GLCM) [65], computed by a 3D analysis of the tumor region with 26-voxel connectivity and simultaneously taking into account the neighboring properties of voxels in all the 3D direction [66], after image quantization on 32 grey levels. The obtained features were energy, contrast, entropy, homogeneity, correlation, sum average, variance, dissimilarity, and auto correlation.

Therefore, considering the first- and second-order features computed for each of the seven images in addition to the shape features, a total of 163 features were obtained.

### 2.8. Multivariable Analysis

Six classification tasks were chosen: ER+/ER−, PR+/PR−, HER2+/HER2−, TN/NTN (non-triple negative, that is, presence of at least one hormonal receptor expression), ki67+/ki67− (using a cut-off of 20%), and lowGrade/highGrade (low G1-G2 and high G3).

The multivariable predictive models were obtained following the method described by Vallières et al. [66], using at each step an imbalance-adjusted bootstrap resampling (IABR) on 1000 samples.

First, for each task, from the large initial set of 163 features, a reduced feature set of 25 features was computed through a stepwise forward feature selection scheme. The first feature was chosen as the best one (i.e., the one that maximized Spearman's rank correlation with the outcome under investigation). Then, one at a time, features were added (up to 25) that maximized a gain equation, given by the linear combination of Spearman's rank correlation (between the feature and the outcome) and the Maximal Information Coefficient (between the feature that was tested and the ones that were yet included in the reduced set) [67].

Then, from the reduced feature set, logistic regression models of order *i* from 1 to 10 that would best predict the outcome under investigation were obtained with another stepwise forward feature selection that, one by one, added to the *i*th model the feature that maximized the 0.632+bootstrap area under the receiver operating characteristic curve (AUC) [68] of the models of order *i*.

Finally, for each classification task, the prediction model was obtained choosing the order that maximize the AUC and computing the final model logistic regression coefficients for the aforementioned combination of feature using IABR.

Mann-Whitney *U* test was used to study the association between each classification task and both the single features of the respective reduced feature sets and the computed prediction models.

## 3. Results

For each of the six classification tasks, the study population, based on the availability of the histological markers under investigation, was reported in Table 1.

The reduced feature sets, one for each classification task, computed according to the stepwise forward feature selection scheme and each composed by the 25 top ranked features in the gain equation, were reported in Table 2, together with the *p* values of the Mann-Whitney *U* test for each feature. At this univariate analysis only median, mean, and energy of *k*^trans^, together with the mean of *k*_ep_, resulted to be significantly associated (considering the Bonferroni adjusted *p* value for multiple comparison) with the ki67+/ki67− outcome.

**Figure 1:**
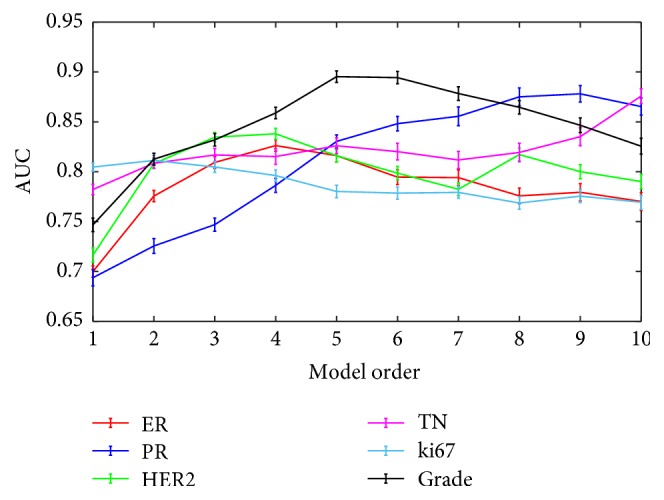
Area under the receiver operating characteristic curve of the multivariable models for each classification task, for model orders from 1 to 10.

For each reduced feature set, multivariable logistic regression models of order from 1 to 10 were obtained and their prediction performance for the different classification tasks was reported in terms of AUC in Figure 1.

By inspecting the curves in Figure 1, the best prediction results were overall reached for classification of tumor grade. Interestingly, for ki67 level discrimination task, which had individually significant features at the Mann-Whitney *U* test (see Table 2), the AUC values did not show any improvement after order 2. For each task, the best model was chosen using as figure of merit the AUC and the selected features were given as input to the logistic regression. The order of the chosen models and the associated prediction performance were reported in Table 3.

The final computation of the multivariable model coefficients led to the following prediction models for ER, PR, HER2+ expression, TN, ki67, and grade, respectively:

(1) gERxi=0.03kep  GLCM  AutoCorrelation−261.80iAUC  GLCM  Variance−18.07iAUC  GLCM  Correlation−6.47postC  GLCM  Entropy+66.96,gPRxi=−1094R1  Uniformity−0.07R1  Mad−36.38kep  GLCM  Correlation+3480kep  GLCM  Sum  Average−0.14iAUC  GLCM  Autocorrelation−16.30R1  Entropy+3837postC  GLCM  Energy+27.09R1  GLCM  Correlation+122.10,gHER2xi=−4.99TIRM  Skeweness−4.69ve  Skeweness+7.01R1  Skeweness+0.01ve  Max−16.66,gTNxi=−0.88R1  GLCM  Autocorrelation−31.79R1  Skeweness+31.44ktrans  GLCM  Entropy+22.98postC  Skeweness−5474postC  GLCM  Energy−0.08TIRM  Mean+18330R1  GLCM  Sum  Average+265R1  GLCM  Homogeneity+1305iAUC  Variance+4.343iAUC  Kurtosis−423.20,gki67xi=ktrans  Energy+0.03kep  Median−1.78,gGradexi=73.73ktrans  GLCM  Homogeneity+20.38ktrans  GLCM  Entropy−0.03TIRM  Max+442.20iAUC  GLCM  Variance+1566TIRM  GLCM  Sum  Average−212.90.

The most recurrent features in the models were skeweness and entropy and, to a lesser extent, auto correlation, variance, correlation, sum average, and energy, while no shape feature was included into the models. Looking at the source images, a greater number of occurrences was found for *R*_1_ map than TIRM images, while the pharmacokinetic maps and the postcontrast acquisition were equally frequent, with the exception of *v*_*e*_ that appeared only once in the prediction models.

**Figure 2:**
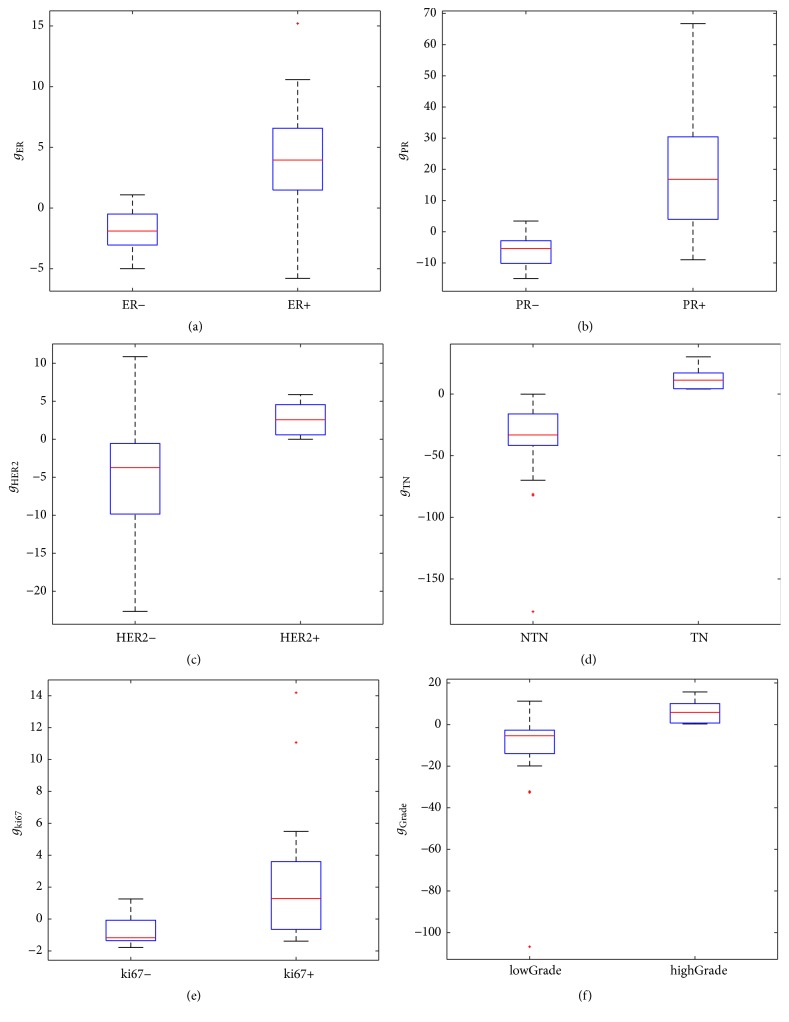
Box plot of the multivariable models obtained for each classification task. From left to right and from top to bottom: (a) ER expression, (b) PR expression, (c) HER2 expression, (d) TN type, (e) ki67 level, and (f) tumor grade.

The Mann-Whitney *U* test revealed a higher discriminative power of the obtained multivariable models compared to the most significant single feature (Table 2), for each classification task (ER expression: *p* value = 0.05 · 10^−2^, PR expression: *p* value = 0.09 · 10^−4^, HER2 expression: *p* value = 0.02 · 10^−3^, TN 0.03 · 10^−2^, ki67 level: *p* value = 0.04 · 10^−3^, and grade: *p* value = 0.02 · 10^−4^). These results are also visible in Figure 2, where, for each classification task, the box plot of the multivariable model was reported.

## 4. Discussion

In this work a radiomic approach to predict different histological outcomes was developed on the basis of a DCE-MRI protocol including pharmacokinetic parametric maps. Six classification tasks were tested, including the molecular receptor status (ER+/ER−, PR+/PR−, HER2+/HER2−, and TN/NTN), ki67 levels, and tumor grade. The molecular receptors are an immunohistochemistry surrogate for breast cancer subtyping and, together with ki67 levels, allow to differentiate lumA, lumB, HER2, and basal-like. Moreover they are fundamental when choosing personalized treatment or the addition of adjuvant chemotherapy to hormone therapy [15].

The obtained results show that radiomic approaches based on pharmacokinetic maps lead to predictive models with a high discriminative power, reaching AUC values above the 80% and accuracy up to 88%.

In order to assess the added value of the radiomic approach, the discriminative power of the single features of the reduced set has been separately evaluated by means of univariate analysis. When looking at these results (Table 2), *p* values of the Mann-Whitney *U* test show that several features are associated with the tumor histological outcome under investigation, but only mean, median, and energy of *k*^trans^, together with mean of *k*_ep_, were found to be significantly associated with the ki67+/ki67 discrimination task. This may be due to the fact that *k*^trans^ and *k*_ep_ are indeed two quantitative pharmacokinetic parameters related to the tumor permeability and vascularization and to medium contrast wash-out; they are clinically used for the differentiation of breast lesions with nonradiomic approaches, and also previous works [59] demonstrated their utility, correlating them with prognosis and TN subtype.

However, the results obtained with the radiomic approach, which is the high-throughput extraction of features, followed by a learning approach for the construction of a predictive models, led to a higher discriminative power, showing that such methods have a great potential to improve quantitative MRI assessment of the tumors.

Other previous works performed radiomic studies on breast cancer, above all to differentiate between subtypes [14, 16, 20, 34, 44, 52–58] with classification performance lower or similar to our results. However, a direct comparison with them was not directly applicable, considering the different populations and imaging approaches.

Interestingly, in the predictive models obtained in this work, the most recurrent features were skeweness and entropy that were indices of randomness, showing the importance of studying the heterogeneity of the tumors. In particular, entropy, even if computed on the first postcontrast image, was already found to be statistically associated to tumor aggressiveness by Li et al. [34]. Skeweness, instead, was found to be predictive for discriminating molecular subtypes by Fan et al. [16] and Sutton et al. [44]. In particular, in this last work, the authors found the skeweness to be significant at three time points on postcontrast MR images, suggesting the pharmacokinetics as a key component in differentiating the subtype.

Interestingly, all these works found a significant association between the outcome and at least one shape feature. Instead, in our study, since the step of feature reduction, they were excluded with the exception of minimum diameter (in the ki67+/ki67 discrimination task) that, however, did not survive in the predictive model. This may be due to a different feature selection algorithm or to the presence of pharmacokinetic-based feature that may be more strongly associated to the outcome than shape features.

Our study propose, by first, the use of pharmacokinetic and relaxometric maps for the radiomic analyses. In particular, in the obtained predictive models, the pharmacokinetic maps, together with postC, were equally represented (with the exception of *v*_*e*_), proving the added value of multiparametric information. Interestingly, much more instances of *R*_1_ features were found compared to the features from TIRM images. *R*_1_ is a parametric map that in DCE time resolved studies is usually used as auxiliary to the computation of pharmacokinetic parameters and is seldom studied by itself, although it could give important information regarding increased vascularity, presence of edema, or necrosis [69, 70]. Our approach, instead, dealing with this parametric map referring to explicit physiological and structural conditions without the use of contrast media, leads to the generation of more discriminative features, compared to the conventional TIRM sequence. This suggests that *R*_1_ map is more suitable to extract textural properties of the tissues.

This study has some limitations: first of all the sample size. A larger study group need to be studied in the feature, to better conduct a radiomic analysis. Moreover, in the computation of pharmacokinetic maps, a population-based AIF was used: this may be a limitation for a quantitative analysis and further evaluation is needed to understand the impact of different AIF on the prediction results. In addition, the diffusion and testing of the obtained models on other populations is limited by the fact that time resolved DCE-MRI protocols for the computation of pharmacokinetic models is not always available in the clinical practice. However, this study paves the way to the study of *R*_1_ map as itself and not necessarily related to the computation of *k*^trans^, *k*_ep_, *v*_*e*_, and iAUC

In conclusion, DCE-MRI pharmacokinetic-based analysis along with *R*_1_ leads to the creation of predictive model that can help in differentiation between molecular receptor status, ki67 levels, and tumor grade with high accuracy. In this direction, further studies will be conducted on the development of models that differentiates between subtypes and including PET images or other MRI acquisition techniques, such as Diffusion Weighted Imaging, and genomic data.

## Figures and Tables

**Table 1:** Sample size and groups for each classification task.

	Total number	Positive	Negative
ER+/ER−	48	40	8
PR+/PR−	48	38	10
HER2+/HER2−	48	12	36
TN(+)/NTN(−)	48	5	43
Ki67+/Ki67−	49	28	21
lowGrade(−)/highGrade(+)	42	14	28

**Table 2:** Reduced feature set of each classification task. For each feature, the image from which it was extracted is indicated (if it is a first- or second-order feature), the feature name, and the *p* value of the Mann-Whitney *U* test. In bold are indicated the features that are significant, according to the Bonferroni correction for multiple comparison.

ER+/ER−	PR+/PR−	HER2+/HER2−	TN/NTN	Ki67+/Ki67−	lowGrade/highGrade
iAUC – GLCM Variance (*p* = 0.05)	*R*_1_ – Std (*p* = 0.06)	*k* ^trans^ – Median (*p* = 0.02)	*k* ^trans^ – GLCM Variance (*p* = 0.03)	**k** ^**trans**^ ** – Median (** **p** ** = 0.**02 · 1**0**^−**2**^**)**	*k* ^trans^ – GLCM Entropy (*p* = 0.08·10^−1^)

*k*_ep_ – GLCM Sum Average (*p* = 0.09)	TIRM – GLCM Entropy (*p* = 0.89)	postC – Skeweness (*p* = 0.05)	*R*_1_ – Skeweness (*p* = 0.04)	*R*_1_ – Energy (*p* = 0.02)	iAUC – GLCM Energy (*p* = 0.02)

postC – Uniformity (*p* = 0.11)	postC – Entropy (*p* = 0.09)	TIRM – Skeweness (*p* = 0.04)	*R*_1_ – GLCM Energy (*p* = 0.05)	*k* ^trans^ – GLCM Entropy (*p* = 0.03)	TIRM – GLCM Sum Average (*p* = 0.16)

TIRM – Mean (*p* = 0.20)	*k*_ep_ – GLCM Sum Average (*p* = 0.19)	*v*_*e*_ – Skeweness (*p* = 0.09)	TIRM – Rms (*p* = 0.17)	**k**_**ep**_ ** – Mean (** **p** ** = 0.0**3 · 1**0**^−**2**^**)**	*k* ^trans^ – GLCM Variance (*p* = 0.07)

*R*_1_ – GLCM Energy (*p* = 0.09)	TIRM – Mean (*p* = 0.22)	postC – Std (*p* = 0.10)	postC – Skeweness (*p* = 0.16)	**k** ^**trans**^ ** – Energy (** **p** ** = 0.0**7 · 1**0**^−**2**^**)**	TIRM – Mad (*p* = 0.10)

*k* ^trans^ – GLCM Variance (*p* = 0.08)	iAUC – Kurtosis (*p* = 0.22)	*R*_1_ – Skeweness (*p* = 0.08)	iAUC – Kurtosis (*p* = 0.10)	**k** ^**trans**^ ** – Mean (** **p** ** = 0.0**3 · 1**0**^−**2**^**)**	*k*_ep_ – Mad (*p* = 0.10)

*k*_ep_ – Skeweness (*p* = 0.13)	*K*_ep_ – GLCM Variance (*p* = 0.25)	*v*_*e*_ – GLCM Entropy (*p* = 0.17)	*R*_1_ – Kurtosis (*p* = 0.06)	*k*_ep_ – Rms (*p* = 0.01 · 10^−1^)	iAUC – GLCM Entropy (*p* = 0.02)

TIRM – Kurtosis (*p* = 0.27)	*R*_1_ – Variance (*p* = 0.06)	*R*_1_ – Std (*p* = 0.15)	*K* ^trans^ – GLCM Contrast (*p* = 0.09)	*k* ^trans^ – Rms (*p* = 0.04 · 10^−2^)	postC – Uniformity (*p* = 0.11)

postC – Entropy (*p* = 0.13)	postC – Uniformity (*p* = 0.09)	*k* ^trans^ – Energy (*p* = 0.04)	*k* ^trans^ – GLCM Dissimilarity (*p* = 0.12)	TIRM – GLCM Entropy (*p* = 0.075)	*K* ^trans^ – GLCM Homogeneity (*p* = 0.05)

*R*_1_ – Skeweness (*p* = 0.17)	*R*_1_ – Mad (*p* = 0.07)	*k* ^trans^ – Rms (*p* = 0.02)	*v*_*e*_ – GLCM Variance (*p* = 0.12)	iAUC – Energy (*p* = 0.09 · 10^−2^)	iAUC – Skeweness (*p* = 0.04)

*v*_*e*_ – GLCM Entropy (*p* = 0.26)	*k*_ep_ – GLCM Correlation (*p* = 0.24)	*k*_ep_ – Mad (*p* = 0.03)	TIRM – Mean (*p* = 0.18)	postC – Skeweness (*p* = 0.12)	*k* ^trans^ – GLCM Energy (*p* = 0.03)

*R*_1_ – GLCM Entropy (*p* = 0.17)	*k*_ep_ – Skeweness (*p* = 0.19)	iAUC – Median (*p* = 0.04)	iAUC – GLCM Variance (*p* = 0.11)	*k*_ep_ – Median (*p* = 0.06 · 10^−2^)	iAUC – Kurtosis (*p* = 0.04)

*K*_ep_ – GLCM AutoCorrelation (*p* = 0.13)	*R*_1_ – Uniformity (*p* = 0.21)	iAUC – Mean (*p* = 0.04)	*k* ^trans^ – GLCM Entropy (*p* = 0.19)	*ve* – GLCM Sum Average (*p* = 0.04)	iAUC – GLCM Variance (*p* = 0.08)

*R*_1_ – GLCM AutoCorrelation (*p* = 0.21)	TIRM – Rms (*p* = 0.19)	*v*_*e*_– Max (*p* = 0.17)	*R*_1_ – GLCM Autocorrelation (*p* = 0.23)	*k* ^trans^ – Max (*p* = 0.01 · 10^−1^)	*K* ^trans^ – GLCM Dissimilarity (*p* = 0.07)

TIRM – Rms (*p* = 0.20)	*R*_1_ – GLCM Correlation (*p* = 0.25)	postC – Max (*p* = 0.09)	postC – GLCM Entropy (*p* = 0.24)	*v*_*e*_ – GLCM Autocorrelation (*p* = 0.04)	*k*_ep_ – GLCM Entropy (*p* = 0.15)

iAUC – Kurtosis (*p* = 0.29)	*R*_1_ – GLCM Energy (*p* = 0.24)	postC – Variance (*p* = 0.10)	TIRM – Median (*p* = 0.20)	*k* ^trans^ – Std (*p* = 0.03 · 10^−1^)	iAUC – Mad (*p* = 0.14)

iAUC – GLCM Correlation (*p* = 0.28)	*R*_1_ – Max (*p* = 0.10)	*R*_1_ – Variance (*p* = 0.14)	TIRM – Kurtosis (*p* = 0.30)	*k* ^trans^ – Variance (*p* = 0.03 · 10^−1^)	postC – Skeweness (*p* = 0.22)

*k* ^trans^ – Skeweness (*p* = 0.27)	iAUC – GLCM Autocorrelation (*p* = 0.23)	iAUC – Energy (*p* = 0.05)	TIRM – GLCM Energy (*p* = 0.23)	*k*_ep_ – GLCM Correlation (*p* = 0.05)	*R*_1_ – GLCM Energy (*p* = 0.17)

TIRM – Median (*p* = 0.24)	*R*_1_ – Kurtosis (*p* = 0.18)	postC – Median (*p* = 0.16)	TIRM – GLCM Variance (*p* = 0.27)	*k*_ep_ – Mad (*p* = 0.03 · 10^−1^)	postC – Entropy (*p* = 0.17)

*R*_1_ – GLCM Sum Average (*p* = 0.28)	TIRM – Std (*p* = 0.23)	*k*_ep_ – Std (*p* = 0.05)	*R*_1_ – GLCM Sum Average (*p* = 0.32)	*k*_ep_ – Entropy (*p* = 0.08 · 10^−2^)	iAUC – Std (*p* = 0.16)

postC – GLCM Entropy (*p* = 0.29)	TIRM – Variance (*p* = 0.23)	*k*_ep_ – Variance (*p* = 0.05)	*K* ^trans^ – GLCM Homogeneity (*p* = 0.23)	postC – Kurtosis (*p* = 0.20)	TIRM – GLCM Autocorrelation (*p* = 0.16)

postC – GLCM Energy (*p* = 0.27)	*R*_1_ – Energy (*p* = 0.20)	*k* ^trans^ – Mean (*p* = 0.03)	*R*_1_ – GLCM Entropy (*p* = 0.34)	Minimun diameter (*p* = 0.08)	TIRM – Max (*p* = 0.12)

*R*_1_ – Kurtosis (*p* = 0.20)	*R*_1_ – GLCM Variance (*p* = 0.25)	*K* ^trans^ – GLCM Variance (*p* = 0.20)	*k*_ep_ – Mad (*p* = 0.35)	*k* ^trans^ – Mad (*p* = 0.04 · 10^−1^)	*k* ^trans^ – Kurtosis (*p* = 0.06)

*K*_ep_ – Mad (*p* = 0.38)	postC – GLCM Energy (*p* = 0.27)	*k*_ep_ – Skeweness (*p* = 0.20)	*R*_1_ – GLCM Homogeneity (*p* = 0.20)	*k*_ep_ – Energy (*p* = 0.04 · 10^−1^)	*k* ^trans^ – Skeweness (*p* = 0.12)

*K*_ep_ – GLCM Variance (*p* = 0.28)	*R*_1_ – Entropy (*p* = 0.22)	postC – Rms (*p* = 0.18)	postC – GLCM Energy (*p* = 0.34)	*R*_1_ – GLCM Correlation (*p* = 0.10)	*R*_1_ – Skeweness (*p* = 0.22)

**Table 3:** Results of multivariable analysis. For each classification task, the model with the higher AUC was chosen and its order, AUC, sensitivity, specificity, and accuracy were reported together with the standard error on a 95% confidence interval over all bootstrap sample.

	Order	AUC	Sensitivity	Specificity	Accuracy
ER+/ER−	4	0.826 ± 0.006	0.833 ± 0.004	0.587 ± 0.016	0.804 ± 0.003
PR+/PR−	8	0.875 ± 0.009	0.895 ± 0.005	0.730 ± 0.019	0.882 ± 0.005
HER2+/HER2−	4	0.838 ± 0.006	0.623 ± 0.014	0.825 ± 0.005	0.785 ± 0.004
TN/NTN	10	0.876 ± 0.007	0.660 ± 0.022	0.896 ± 0.004	0.881 ± 0.004
Ki67+/Ki67−	2	0.811 ± 0.005	0.641 ± 0.006	0.736 ± 0.007	0.677 ± 0.004
lowGrade/highGrade	5	0.895 ± 0.006	0.735 ± 0.012	0.865 ± 0.006	0.807 ± 0.004

## References

[1] Siegel R. L., Miller K. D., Jemal A. (2016). Cancer statistics, 2016. *CA: A Cancer Journal for Clinicians*.

[2] Torre L. A., Siegel R. L., Ward E. M., Jemal A. (2016). Global cancer incidence and mortality rates and trends—an update. *Cancer Epidemiology, Biomarkers & Prevention*.

[3] Zimmer A., Gatti-Mays M., Soltani S. (2017). Abstract PD6-01: Analysis of breast cancer in young women in the department of defense (DOD) database. *American Association for Cancer Research*.

[4] van't Veer L. J., Dai H., van de Vijver M. J. (2002). Gene expression profiling predicts clinical outcome of breast cancer. *Nature*.

[5] Sørlie T., Perou C. M., Tibshirani R. (2001). Gene expression patterns of breast carcinomas distinguish tumor subclasses with clinical implications. *Proceedings of the National Acadamy of Sciences of the United States of America*.

[6] Huber K. E., Carey L. A., Wazer D. E. (2009). Breast cancer molecular subtypes in patients with locally advanced disease: impact on prognosis, patterns of recurrence, and response to therapy. *Seminars in Radiation Oncology*.

[7] Goldhirsch A., Wood W. C., Coates A. S., Gelber R. D., Thürlimann B., Senn H.-J. (2011). Strategies for subtypes-dealing with the diversity of breast cancer: highlights of the St Gallen international expert consensus on the primary therapy of early breast cancer 2011. *Annals of Oncology*.

[8] Geyer F. C., Rodrigues D. N., Weigelt B., Reis-Filho J. S. (2012). Molecular classification of estrogen receptor-positive/luminal breast cancers. *Advances in Anatomic Pathology*.

[9] Cancer Genome Atlas Network (2012). Comprehensive molecular portraits of human breast tumours. *Nature*.

[10] Bagaria S. P., Ray P. S., Sim M.-S. (2014). Personalizing breast cancer staging by the inclusion of ER, PR, and HER2. *JAMA Surgery*.

[11] Perou C. M., Sørile T., Eisen M. B. (2000). Molecular portraits of human breast tumours. *Nature*.

[12] Harbeck N., Thomssen C., Gnant M. (2013). St. Gallen 2013: brief preliminary summary of the consensus discussion. *Breast Care*.

[13] Bustreo S., Osella-Abate S., Cassoni P. (2016). Optimal Ki67 cut-off for luminal breast cancer prognostic evaluation: a large case series study with a long-term follow-up. *Breast Cancer Research and Treatment*.

[14] Sutton E. J., Oh J. H., Dashevsky B. Z. (2015). Breast cancer subtype intertumor heterogeneity: MRI-based features predict results of a genomic assay. *Journal of Magnetic Resonance Imaging*.

[15] Cheang M. C. U., Chia S. K., Voduc D. (2009). Ki67 index, HER2 status, and prognosis of patients with luminal B breast cancer. *Journal of the National Cancer Institute*.

[16] Fan M., Li H., Wang S., Zheng B., Zhang J., Li L. (2017). Radiomic analysis reveals DCE-MRI features for prediction of molecular subtypes of breast cancer. *PLoS ONE*.

[17] Metzger-Filho O., Sun Z., Viale G. (2013). Patterns of recurrence and outcome according to breast cancer subtypes in lymph node-negative disease: Results from international breast cancer study group trials VIII and IX. *Journal of Clinical Oncology*.

[18] Esteva F. J., Yu D., Hung M.-C., Hortobagyi G. N. (2010). Molecular predictors of response to trastuzumab and lapatinib in breast cancer. *Nature Reviews Clinical Oncology*.

[19] Kontzoglou K., Palla V., Karaolanis G. (2013). Correlation between Ki67 and Breast Cancer Prognosis. *Oncology (Switzerland)*.

[20] Wang J., Kato F., Oyama-Manabe N. (2015). Identifying triple-negative breast cancer using background parenchymal enhancement heterogeneity on dynamic contrast-enhanced MRI: A pilot radiomics study. *PLoS ONE*.

[21] Egner J. R. (2010). AJCC Cancer Staging Manual. *Journal of the American Medical Association*.

[22] Zaha D. C. (2014). Significance of immunohistochemistry in breast cancer. *World Journal of Clinical Oncology*.

[23] Bruening W., Fontanarosa J., Tipton K., Treadwell J. R., Launders J., Schoelles K. (2010). Systematic review: Comparative effectiveness of core-needle and open surgical biopsy to diagnose breast lesions. *Annals of Internal Medicine*.

[24] Tamaki K. K., Sasano H., Ishida T. (2010). Comparison of core needle biopsy (CNB) and surgical specimens for accurate preoperative evaluation of ER, PgR and HER2 status of breast cancer patients. *Cancer Science*.

[25] Ough M., Velasco J., Hieken T. J. (2011). A comparative analysis of core needle biopsy and final excision for breast cancer: Histology and marker expression. *The American Journal of Surgery*.

[26] Longo D. L. (2012). Tumor heterogeneity and personalized medicine. *The New England Journal of Medicine*.

[27] Gatenby R. A., Grove O., Gillies R. J. (2013). Quantitative imaging in cancer evolution and ecology. *Radiology*.

[28] Gillies R. J., Anderson A. R., Gatenby R. A., Morse D. L. (2010). The biology underlying molecular imaging in oncology: from genome to anatome and back again. *Clinical Radiology*.

[29] Aerts H. J., Velazquez E. R., Leijenaar R. T. (2014). Decoding tumour phenotype by noninvasive imaging using a quantitative radiomics approach. *Nature Communications*.

[30] Incoronato M., Aiello M., Infante T. (2017). Radiogenomic Analysis of Oncological Data: A Technical Survey. *International Journal of Molecular Sciences*.

[31] Diehn M., Nardini C., Wang D. S. (2008). Identification of noninvasive imaging surrogates for brain tumor gene-expression modules. *Proceedings of the National Acadamy of Sciences of the United States of America*.

[32] Segal E., Sirlin C. B., Ooi C. (2007). Decoding global gene expression programs in liver cancer by noninvasive imaging. *Nature Biotechnology*.

[33] Gillies R. J., Kinahan P. E., Hricak H. (2016). Radiomics: images are more than pictures, they are data. *Radiology*.

[34] Li H., Zhu Y., Burnside E. S. (2016). Quantitative MRI radiomics in the prediction of molecular classifications of breast cancer subtypes in the TCGA/TCIA data set. *NPJ Breast Cancer*.

[35] Yip S. S. F., Aerts H. J. W. L. (2016). Applications and limitations of radiomics. *Physics in Medicine and Biology*.

[36] Kirienko M., Cozzi L., Antunovic L. (2017). Prediction of disease-free survival by the PET/CT radiomic signature in non-small cell lung cancer patients undergoing surgery. *European Journal of Nuclear Medicine and Molecular Imaging*.

[37] Blanc-Durand P. (2017). 18F-FDG PET-based Radiomics Score Predicts Survival in Patients treated with Yttrium-90 Transarterial Radioembolization for Unresectable Hepatocellular Carcinoma. *Journal of Nuclear Medicine*.

[38] Wang J., Wu C.-J., Bao M.-L., Zhang J., Wang X.-N., Zhang Y.-D. (2017). Machine learning-based analysis of MR radiomics can help to improve the diagnostic performance of PI-RADS v2 in clinically relevant prostate cancer. *European Radiology*.

[39] Jochems A., Hoebers F., De Ruysscher D. (2017). EP-1605: Deep learning of radiomics features for survival prediction in NSCLC and Head and Neck carcinoma. *Radiotherapy & Oncology*.

[40] Ingrisch M., Schneider M. J., Nörenberg D. (2017). Radiomic analysis reveals prognostic information in T1-weighted baseline magnetic resonance imaging in patients with glioblastoma. *Investigative Radiology*.

[41] Zhang B., Tian J., Dong D. (2017). Radiomics Features of Multiparametric MRI as Novel Prognostic Factors in Advanced Nasopharyngeal Carcinoma. *Clinical Cancer Research*.

[42] Pinker K., Shitano F., Sala E. (2017). Background, current role, and potential applications of radiogenomics. *Journal of Magnetic Resonance Imaging*.

[43] Gallivanone F., Panzeri M. M., Canevari C. (2017). Biomarkers from in vivo molecular imaging of breast cancer: pretreatment 18F-FDG PET predicts patient prognosis, and pretreatment DWI-MR predicts response to neoadjuvant chemotherapy. *Magnetic Resonance Materials in Physics, Biology and Medicine*.

[44] Sutton E. J., Dashevsky B. Z., Oh J. H. (2016). Breast cancer molecular subtype classifier that incorporates MRI features. *Journal of Magnetic Resonance Imaging*.

[45] Hylton N. (2006). Dynamic contrast-enhanced magnetic resonance imaging as an imaging biomarker. *Journal of Clinical Oncology*.

[46] Bhooshan N., Giger M. L., Jansen S. A., Li H., Lan L., Newstead G. M. (2010). Cancerous breast lesions on dynamic contrast-enhanced MR images: computerized characterization for image-based prognostic markers. *Radiology*.

[47] Bhooshan N., Giger M., Edwards D. (2011). Computerized three-class classification of MRI-based prognostic markers for breast cancer. *Physics in Medicine and Biology*.

[48] Braman N. M., Etesami M., Prasanna P. (2017). Erratum to: Intratumoral and peritumoral radiomics for the pretreatment prediction of pathological complete response to neoadjuvant chemotherapy based on breast DCE-MRI. *Breast Cancer Research*.

[49] Wu J., Gong G., Cui Y., Li R. (2016). Intratumor partitioning and texture analysis of dynamic contrast-enhanced (DCE)-MRI identifies relevant tumor subregions to predict pathological response of breast cancer to neoadjuvant chemotherapy. *Journal of Magnetic Resonance Imaging*.

[50] Fan M., Wu G., Cheng H., Zhang J., Shao G., Li L. (2017). Radiomic analysis of DCE-MRI for prediction of response to neoadjuvant chemotherapy in breast cancer patients. *European Journal of Radiology*.

[51] Zhu Y., Li H., Guo W. (2015). Deciphering genomic underpinnings of quantitative MRI-based radiomic phenotypes of invasive breast carcinoma. *Scientific Reports*.

[52] Li H., Zhu Y., Burnside E. S. (2016). MR imaging radiomics signatures for predicting the risk of breast cancer recurrence as given by research versions of MammaPrint, oncotype DX, and PAM50 gene assays. *Radiology*.

[53] Agner S. C., Rosen M. A., Englander S. (2014). Computerized image analysis for identifying triple-negative breast cancers and differentiating them from other molecular subtypes of breast cancer on dynamic contrast-enhanced mr images: A feasibility study. *Radiology*.

[54] Mazurowski M. A., Zhang J., Grimm L. J., Yoon S. C., Silber J. I. (2014). Radiogenomic analysis of breast cancer: Luminal B molecular subtype is associated with enhancement dynamics at MR imaging. *Radiology*.

[55] Grimm L. J., Zhang J., Mazurowski M. A. (2015). Computational approach to radiogenomics of breast cancer: Luminal A and luminal B molecular subtypes are associated with imaging features on routine breast MRI extracted using computer vision algorithms. *Journal of Magnetic Resonance Imaging*.

[56] Guo W., Li H., Zhu Y. (2015). Prediction of clinical phenotypes in invasive breast carcinomas from the integration of radiomics and genomics data. *Journal of Medical Imaging*.

[57] Blaschke E., Abe H. (2015). MRI phenotype of breast cancer: Kinetic assessment for molecular subtypes. *Journal of Magnetic Resonance Imaging*.

[58] Yamaguchi K., Abe H., Newstead G. M. (2015). Intratumoral heterogeneity of the distribution of kinetic parameters in breast cancer: comparison based on the molecular subtypes of invasive breast cancer. *Breast Cancer*.

[59] Koo H. R., Cho N., Song I. C. (2012). Correlation of perfusion parameters on dynamic contrast-enhanced MRI with prognostic factors and subtypes of breast cancers. *Journal of Magnetic Resonance Imaging*.

[60] Li Z., Ai T., Hu Y. (2017). Application of whole‐lesion histogram analysis of pharmacokinetic parameters in dynamic contrast‐enhanced MRI of breast lesions with the CAIPIRINHA‐Dixon‐TWIST‐VIBE technique. *Journal of Magnetic Resonance Imaging*.

[61] Fedorov A., Beichel R., Kalpathy-Cramer J. (2012). 3D slicer as an image computing platform for the quantitative imaging network. *Magnetic Resonance Imaging*.

[62] Li X., Arlinghaus L. R., Ayers G. D. (2014). DCE-MRI analysis methods for predicting the response of breast cancer to neoadjuvant chemotherapy: Pilot study findings. *Magnetic Resonance in Medicine*.

[63] Tofts P. S. (2010). T1-weighted DCE imaging concepts: modelling, acquisition and analysis. *Signal*.

[64] Collewet G., Strzelecki M., Mariette F. (2004). Influence of MRI acquisition protocols and image intensity normalization methods on texture classification. *Magnetic Resonance Imaging*.

[65] Haralick R. M., Shanmugam K., Dinstein I. (1973). Textural features for image classification. *IEEE Transactions on Systems, Man, and Cybernetics*.

[66] Vallières M., Freeman C. R., Skamene S. R., El Naqa I. (2015). A radiomics model from joint FDG-PET and MRI texture features for the prediction of lung metastases in soft-tissue sarcomas of the extremities. *Physics in Medicine and Biology*.

[67] Reshef D. N., Reshef Y. A., Finucane H. K. (2011). Detecting novel associations in large data sets. *Science*.

[68] Sahiner B., Chan H.-P., Hadjiiski L. (2008). Classifier performance prediction for computer-aided diagnosis using a limited dataset. *Medical Physics*.

[69] Juntu J., Sijbers J., De Backer S., Rajan J., Van Dyck D. (2010). Machine learning study of several classifiers trained with texture analysis features to differentiate benign from malignant soft-tissue tumors in T1-MRI images. *Journal of Magnetic Resonance Imaging*.

[70] Monti S., Borrelli P., Tedeschi E., Cocozza S., Palma G. (2017). RESUME: Turning an SWI acquisition into a fast qMRI protocol. *PLoS ONE*.

